# Supplementary material for: Stated preferences for new HIV prevention technologies among men who have sex with men in India: A discrete choice experiment
Source: PLoS One. 2024 Jul 24;19(7):e0289396. doi: 10.1371/journal.pone.0289396 (PMC11268640; doi:10.1371/journal.pone.0289396)
Supplement: S1 Appendix — (DOCX) [file pone.0289396.s001.docx]

**Appendix**: **Descriptions presented of HIV prevention products**

A **rectal microbicide** would be in the form of a topical lubricant or gel. It would be put in the anus to prevent or reduce a person’s risk of HIV infection from anal sex. Rectal microbicides are currently being tested but are not available at present.

An **HIV vaccine** would be given as a shot (injection). It strengthens the body’s (immune) protection system to prevent or reduce a person’s risk of HIV infection. HIV vaccines are currently being tested but are not available at present.

**Pre-Exposure Prophylaxis (PrEP)** would be given as a pill. It involves the use of HIV medications by people who do not have HIV to prevent or reduce a person’s risk of HIV infection. Pre-Exposure Prophylaxis (PrEP) is currently licensed for use in the U.S. and some other countries, but not in India.
