# Supplementary material for: Stated preferences for new HIV prevention technologies among men who have sex with men in India: A discrete choice experiment
Source: PLoS One. 2024 Jul 24;19(7):e0289396. doi: 10.1371/journal.pone.0289396 (PMC11268640; doi:10.1371/journal.pone.0289396)
Supplement: Attachment — Submitted filename: Response to Reviewers.docx [file pone.0289396.s003.docx]

**Article ID:** PONE-D-23-22064

**Title:** Stated Preferences for New HIV Prevention Technologies among Men Who have Sex with Men in India: A Discrete Choice Experiment

**Journal Requirements:**

When submitting your revision, we need you to address these additional requirements.

**1.** Please ensure that your manuscript meets PLOS ONE's style requirements, including those for file naming. The PLOS ONE style templates can be found at

<https://journals.plos.org/plosone/s/file?id=wjVg/PLOSOne_formatting_sample_main_body.pdf> and

<https://journals.plos.org/plosone/s/file?id=ba62/PLOSOne_formatting_sample_title_authors_affiliations.pdf>

***Response:*** We have reviewed the manuscript and confirm that it meets PLOS ONE’s style requirements.

**2.** Note from Emily Chenette, Editor in Chief of PLOS ONE, and Iain Hrynaszkiewicz, Director of Open Research Solutions at PLOS: Did you know that depositing data in a repository is associated with up to a 25% citation advantage (https://doi.org/10.1371/journal.pone.0230416)? If you’ve not already done so, consider depositing your raw data in a repository to ensure your work is read, appreciated and cited by the largest possible audience. You’ll also earn an Accessible Data icon on your published paper if you deposit your data in any participating repository (<https://plos.org/open-science/open-data/#accessible-data>).

**3 - 1)** Please provide additional information regarding the considerations made for the participants included in this study. For instance, please discuss whether participants were able to opt out of the study and whether individuals who did not participate receive the same treatment offered to participants.

***Response:*** We now provide further details in the manuscript text regarding these considerations at the beginning of the Methods section.

Page 6, paragraph 2:

“Participation was completely voluntary. Individuals who received an invitation to participate could choose whether or not to visit the community organization to do so. Participants who initiated the questionnaire could also opt out of completion with no penalty; however, since all data were anonymized, their responses could not be removed after survey completion, as explained in the informed consent. All individuals had access to HIV prevention services from the partner CBOs whether or not they chose to participate in the study.”

**3 - 2)** Please include a complete copy of PLOS’ questionnaire on inclusivity in global research in your revised manuscript. Our policy for research in this area aims to improve transparency in the reporting of research performed outside of researchers’ own country or community. The policy applies to researchers who have travelled to a different country to conduct research, research with Indigenous populations or their lands, and research on cultural artefacts. The questionnaire can also be requested at the journal’s discretion for any other submissions, even if these conditions are not met. Please find more information on the policy and a link to download a blank copy of the questionnaire here: <https://journals.plos.org/plosone/s/best-practices-in-research-reporting>. Please upload a completed version of your questionnaire as Supporting Information when you resubmit your manuscript

***Response:*** As requested, we have uploaded a completed version of the questionnaire on inclusivity in global research.

**4.** Thank you for stating the following in the Competing Interests section:

"I have read the journal’s policy and the authors of this manuscript have the following competing interests: PAN reports serving as an Academic Editor for PLOS ONE. All other authors reported no conflicts of interest."

Please confirm that this does not alter your adherence to all PLOS ONE policies on sharing data and materials, by including the following statement: "This does not alter our adherence to  PLOS ONE policies on sharing data and materials.” (as detailed online in our guide for authors <http://journals.plos.org/plosone/s/competing-interests>).  If there are restrictions on sharing of data and/or materials, please state these. Please note that we cannot proceed with consideration of your article until this information has been declared.

Please include your updated Competing Interests statement in your cover letter; we will change the online submission form on your behalf.

***Response:***

As requested, we have included an updated Competing Interests statement in our cover letter, which includes the following text:

“This does not alter our adherence to PLOS ONE policies on sharing data and materials.”

**5.** We note that you have indicated that there are restrictions to data sharing for this study. PLOS only allows data to be available upon request if there are legal or ethical restrictions on sharing data publicly. For more information on unacceptable data access restrictions, please see <http://journals.plos.org/plosone/s/data-availability#loc-unacceptable-data-access-restrictions>.

Before we proceed with your manuscript, please address the following prompts:

**a**) If there are ethical or legal restrictions on sharing a de-identified data set, please explain them in detail (e.g., data contain potentially identifying or sensitive patient information, data are owned by a third-party organization, etc.) and who has imposed them (e.g., a Research Ethics Committee or Institutional Review Board, etc.). Please also provide contact information for a data access committee, ethics committee, or other institutional body to which data requests may be sent.

**b)** If there are no restrictions, please upload the minimal anonymized data set necessary to replicate your study findings to a stable, public repository and provide us with the relevant URLs, DOIs, or accession numbers. For a list of recommended repositories, please see

<https://journals.plos.org/plosone/s/recommended-repositories>. You also have the option of uploading the data as Supporting Information files, but we would recommend depositing data directly to a data repository if possible.

We will update your Data Availability statement on your behalf to reflect the information you provide.

***Response:*** Data are owned by the Humsafar Trust, and the Centre for Sexuality and Health Research and Policy, in India. Due to the sensitivity of participant information about sexuality and HIV in India, the Institutional Review Board (IRB) of the Humsafar Trust has imposed restrictions on public sharing of the data sets. Requests for access to de-identified data sets for the results presented will be granted on a case-by-case basis by contacting the IRB Coordinator of the Humsafar Trust, Mr. Sandeep Mane, at [info@humsafar.org](mailto:info@humsafar.org) or +91-9892940966.

**Additional Editor Comments:**

Two reviewers have revised your manuscript. One of them has several doubts with the selection of attributes and levels. You may estimate your models using the continuous variables where possible, instead using transformations with dummies. For example, you can icroproate to the model the attribute efficacy as a continuous variable (50%, 99%) and to consider a quadratic form for this attribute.

***Response:*** We appreciate this concern, however all attributes in the DCE took on only two values. So, while estimating the models using continuous rather than categorical variables is possible, the re-estimation would be functionally identical. For example, efficacy took on two levels (50% and 99%). If you divide the coefficient estimate from the current model estimations by 49, then you obtain the marginal effect for a 1-percentage-point increase in efficacy. Also, because efficacy takes on only two values, a model with a quadratic function in efficacy cannot be estimated as the efficacy and efficacy^2 variables will be collinear. Thus we have not changed this in the text; however, in deference to Reviewer 2 we now indicate the use of categorical variables with only 2 levels as a potential study limitation, in addition to potential changes in the relevant attributes of products still in the development pipeline. We also have added substantial details about the process of selecting attributes and levels, as in response to Reviewer 2, comment 2.

Page 2, paragraph 3:

“It is also an inherent limitation of DCEs that the preferences revealed are contingent on the product attributes and values modeled. The present study’s use of categorical variables with two levels of each attribute was based on formative qualitative research and pilot DCEs with the study population, following best practices in the development of the DCEs. Although relevant attributes and levels of products in the development pipeline may change as they near approval, this is commensurate with our aim to prepare for future implementation of HIV prevention products in the development pipeline, a benefit of DCE methods.”

Page 9, paragraph 3 – page 10, paragraph 4:

“We used a multistage process to develop the DCEs, in accordance with best practice guidelines [12]. First, we conducted a literature review and formative qualitative research on each prevention technology with the focal study population [7,30,31]. Based on this information, we identified the most salient attributes for these HIV prevention technologies: concerns about the degree of efficacy, dosing frequency, and side effects emerged across our qualitative research on hypothetical HIV vaccines, microbicides, and PrEP [7,30,31]. Additionally, MSM expressed concerns about stigma if it were disclosed that they were accessing these HIV prevention products, such as being judged as sexually “promiscuous” or being “outed” to their families as MSM. This invoked the potential importance of the venue for accessing HIV prevention products as a determinant of product acceptability and use.

Second, we conducted a pilot DCE study with MSM (n = 16) recruited from CBO partners in India; this provided a priori guidance on the model coefficients and guided the selection of attributes (e.g., efficacy) and levels (e.g., 50% vs 99%) for each product in the DCE [32]. It also supported the feasibility of implementing DCEs with this population.

Third, we incorporated methodological recommendations for choice elicitation tasks [32]: alternative attribute levels (e.g., 50% vs 99% efficacy; none vs. minor side effects) need to be sufficiently distinct to be comprehensible to participants, most of whom are unlikely to be able to discern the difference between 50% and 65% efficacy, for example, or a 15% vs. 25% chance of fever or headaches. Our recruitment aimed to reach vulnerable populations of largely low socioeconomic status MSM, many without college education and with limited economic opportunities, a substantial proportion of whom rely on sex work for income. This underscored the importance of selecting attribute levels to facilitate comprehension among participants with low numeracy.

Finally, the very high efficacy of oral PrEP in clinical trials informed our use of 99% as a level. As a result, 99% efficacy was used as the upper value for all 3 products, in contrast with 50% to clearly signify partial efficacy.”

**REVIEWER #1**

The paper focuses on the (hypothetical) willingness to use three HIV prevention technologies among men who have sex with men in India. It represents a comprehensive and methodologically sound study with relevant findings, such as the variability of preferences based on sociodemographic and risk behavior factors. However, some aspects could benefit from refinement for enhanced clarity:

**Comment 1:** The current presentation of the study design is difficult to follow. While it seems that all necessary details regarding recruitment, study design, and the DCE are included, they are not presented in a cohesive and comprehensible manner. For example, one may wonder how the respondents were sampled or how the DCE was designed, only to get an explanation in later sections. This limits the flow of reading considerably. I recommend fundamentally rewriting and reorganizing the sections on study design, sample and data collection, and DCE development to improve clarity and coherence.

***Response:*** We have now substantially rewritten and reorganized the Methods section, which has improved the flow of reading. In accordance with the reviewer’s concern, we first describe the overall Study Design and Context, then include a separate section on Sampling and Recruitment, and thereafter we provide details about the DCE Experimental Design and DCE Development. This helps to clarify the new section on Data Collection, and then Measures, which come afterward. This reorganization also provides additional descriptions to address the Reviewer’s comments 2, 3 and 4 below.

**Comment 2:** The description of the process for assigning individuals to one of the three products is ambiguous and requires further explanation. It remains unclear whether the three products are linked to the three different versions of the questionnaire.

***Response:*** We now provide a clearer description of the assignment of individuals to one of three survey versions, each with a DCE of one product. The three survey versions were the same except for the target product of the DCE.

Page 11, paragraph 3 – page 12, paragraph 1:

“To mitigate potential respondent fatigue, we created three versions of the questionnaire, each of which was identical except for the DCE. Participants were randomly assigned to one of the three versions including a DCE for one prevention technology. The DCE experimental design (based on Bayesian D-error minimization) contained 32 choice scenarios of five hypothetical product alternatives for each of the three products. To reduce cognitive burden on participants, these were each blocked in four groups of eight choice scenarios. Random allocation to survey versions and blocking within each product DCE were programmed on the Tablet devices.”

**Comment 3:** The use of chain-referral sampling raises concerns about the potential for selection bias and survey falsification. I recommend that the authors discuss any quality control measures implemented to ensure that respondents genuinely met the inclusion criteria. To my understanding, there's a risk that participants might falsely have identified as part of the target group to access the study incentives. Addressing this concern helps validating the study's findings as well as strength the paper.

***Response:*** As the reviewer indicates, there are risks in chain-referral sampling, including misrepresentation of one’s eligibility and duplicate participation. We now describe several quality control measures that we implemented to address these concerns. Benefits of the chain-referral method are that it enables one to reach potential participants beyond CBO clientele—often those recruited for HIV research among community samples—who may be at greater risk for HIV due to lack of engagement with HIV education and prevention services. As we now describe in the manuscript

Page 8, paragraph 2:

“We employed several quality control measures to mitigate risks in chain-referral sampling; for example, individuals might present at the study site, having not been referred, and may misrepresent their sexual identity or behavior to enable them to receive study honoraria. For one, peer recruiters were employed by existing CBOs and well-established in the MSM community and recruitment sites; and they were trained to assess the eligibility of potential participants. Second, each recruiter maintained a log register that tracked individuals referred by seeds—directly invited by the peer recruiter—and then cross-checked whether the same individuals were also referred by other seeds (based on duplication in names, ages, and phone numbers in the logs). Third, pre-numbered coupons were issued to all participants, each with a unique identification number (UID), to invite others in the chain-referral process. Peer recruiters tracked new participants with these coupons, thus indicating the recruiter for each individual. Absent an initial invitation as a seed, or a coupon, other individuals were not able to participate. These measures facilitated the chain-referral process and prevented misrepresentation of one’s eligibility and duplicate enrollment.”

**Comment 4:** The burden of eight choice scenarios, each including five cards with different attribute level combinations, seems considerable and could lead to participation fatigue. This fatigue might affect the reliability of responses. I suggest that the authors control for effects of survey fatigue, possibly by focusing on a smaller set of decisions made by each respondent, such as only the first 3-4 decisions, as a robustness check.

***Response:*** It is reasonable to be concerned about respondent fatigue. In fact, a substantial literature on discrete choice experiments, including multiple studies and reviews, has established that respondent fatigue sets in well beyond the eight scenarios that we presented to our participants. See, for example, Meyerhoff et al. (2015) and Hess et al. (2012) [as well as Hensher (2006), and Hensher et al. (2001)]. Several previous feasibility studies on the use of stated preference methods for HIV prevention technologies that similarly use eight scenarios also support this finding. In deference to the reviewer’s concern, we have added a statement and references to the Methods supporting the use of eight choice scenarios.

“The design of the instrument follows WHO guidelines for DCEs [28], using methods we have previously implemented with MSM in Thailand [32]. Previous stated preference research supports participants’ capacity to rate eight choice scenarios, with respondent fatigue occurring well beyond this threshold [33,34]. Moreover, in earlier feasibility studies of using stated preference methods for HIV prevention technologies with eight choice scenarios among several key populations at risk for HIV, the majority of participants indicated the task was easy/somewhat easy to complete, with some describing the choice scenarios as more engaging than a series of individual Likert-type items [35].”

**References**

[33] Hess S, Hensher DA, Daly A (2012) Not bored yet—revisiting respondent fatigue in stated choice experiments. Transportation research part A, 626–644.

[34] Meyerhoff J., M. Oehlmann and P. Weller, (2015) The Influence of Design Dimensions on Stated Choices in an Environmental Context, Environmental and Resource Economics, 61: 385-407.

[35] Lee, S. J., Newman, P. A., Comulada, W. S., Cunningham, W. E., & Duan, N. (2012). Use of conjoint analysis to assess HIV vaccine acceptability: Feasibility of an innovation in the assessment of consumer health-care preferences. International Journal of STDs & AIDS, 23(4), 235–241.

Hensher DA, Stopher PA, Louviere JJ (2001) An exploratory analysis of the effect of numbers of choice sets in designed choice experiments: an airline choice application. J Air Transp Manage 7:373–379.

Hensher DA (2006) Revealing differences in willingness to pay due to the dimensionality of stated choice designs: an initial assessment. Environ Resour Econ 34:7–44.

**Comment 5:** Ideally, the authors would make their Stata code and the choice scenarios created in Ngene available as part of the supplement so that other researchers can build on their work.

***Response:*** We now indicate that the Stata code and Ngene scenarios are available upon request from the lead organization in the study. As we explain, this is due to restrictions from the Institutional Review Board in India on making such data publicly available.

**REVIEWER #2**

**Major issues to be addressed:**
**1)** Improve manuscript to present a review of economic valuation literature regarding DCE and HIV prevention. There is a systematic review of 2021 in the Patient-patient centered outcomes research Journal, and in many others like the Journal of International Aids Society or more general like the Journal of Choice Modelling. Methods and results must be compared and discussed taking into account previous literature.

***Response:*** We have added a paragraph to the Introduction section, in which we discuss the systematic review (n=84 studies) published in *Patient* (Beckham et al., 2021), as suggested by the reviewer, and a previous review in *PLoS One* (n = 57 studies) (Humphrey et al., 2019) that address the use of stated preference methods in the field of HIV. These reviews highlight the dearth of studies using stated preference methods that have been conducted in Asia, and among key populations at risk for HIV, as well as that few studies consider more than one HIV prevention tool within the same population.

Page 5, paragraph 3 – page 6, paragraph 1:

“A number of studies internationally have explored preferences for HIV prevention tools and their product characteristics. In a large systematic review, Beckham et al. [12] indicated a substantial increase in the number of published studies eliciting preferences for HIV prevention tools. A variety of methods for preference elicitation were employed, including conjoint analysis, ‘willingness-to-try’, discrete choice experiments, and contingent valuation. Of the 84 studies included in their review, nearly 90% were conducted in either sub-Saharan Africa (54%) or North America (36%), with only 10 studies in Asia (six of which were conducted in Thailand). Only 54% of studies included key populations at high risk for HIV (e.g., sex workers, MSM). An earlier review [13] similarly found that most stated preference research on HIV prevention and treatment more generally had been concentrated on populations in North America, Europe, and Africa, with only about one-quarter of research studies overall conducted on key populations such as MSM, female sex workers, and transgender women. In preference studies for HIV prevention tools, key populations at risk, particularly in Asia, have been understudied. Moreover, few studies have considered preferences for multiple HIV prevention tools within the same population.”

In the Methods section, we have added a sentence that specifies our design and that it is a widely accepted approach in the design of DCE (Johnson et al., 2013). We also refer to previous stated preference studies on HIV (Rodriquez & Mitchell, 2023).

Page 9, paragraph 2:

“The DCE design follows widely accepted approaches for DCEs in health applications [14], including methods for selecting the attributes and values modeled [12]. Specifically, the design was developed using a Bayesian D-error minimizing approach with dummy coded variables obtained using Ngene software (ChoiceMetrics, Sydney, Australia).”

Page 7, paragraph 1:

“DCEs are a now established methodology for quantitively estimating end-user preferences in healthcare [14]. Studies have increasingly applied DCEs to discern preferences for HIV prevention, including new prevention technologies, among vulnerable populations [12,15].”

In the Results section, we discuss all main findings regarding the attributes in the context of previous literature.

**References**

[12] Beckham SW, Crossnohere NL, Gross M, Bridges JF. Eliciting preferences for HIV prevention technologies: A systematic review. Patient. 2021;14(2):151-174.

[13] Humphrey JM, Naanyu V, MacDonald KR, Wools-Kaloustian K, Zimet GD. Stated-preference research in HIV: a scoping review. PloS One. 2019;14(10):e0224566.

[14] Johnson RF, Lancsar E, Marshall D, Kilambi V, Mühlbacher A, Regier DA, et al. Constructing experimental designs for discrete-choice experiments: Report of the ISPOR Conjoint Analysis Experimental Design Good Research Practices Task Force. Value Health. 2013;16(1):3-13.

[15] Rodriguez CA, Mitchell JW. Use of stated preference methods in HIV treatment and prevention research in the United States: A systematic review. AIDS Behav. 2023;27:2328-2359.

**2)** Authors must explain the process followed to select attributes and levels. Levels of some crucial attributes (like efficacy, 50%, 99%; side effects, none, minor) may have conditioned their high significance in the analysis. Also, it would be important to see the information provided before the choice, to check the scenario and previous information, which could be crucial for respondents to fully understand the task.

***Response:***

As requested by the reviewer, we have added details about the multistage process of selecting attributes and levels to model in the DCEs, and additional citations to support our process. In deference to the reviewer’s concern, we also indicate in the Limitations section that the modeling of other attributes and levels might result in different results and preference profiles; nevertheless, we followed a rigorous process based on formative research and pilot testing of the DCEs with the focal population for the study. As requested, we have also included the English-language translations of the brief introductory description of each product as presented to participants before conducting the DCE, added as Appendix A.

Page 9, paragraph 3 – page 10, paragraph 4:

“We used a multistage process to develop the DCEs, in accordance with best practice guidelines [12]. First, we conducted a literature review and formative qualitative research on each prevention technology with the focal study population [7,30,31]. Based on this information, we identified the most salient attributes for these HIV prevention technologies: concerns about the degree of efficacy, dosing frequency, and side effects emerged across our qualitative research on hypothetical HIV vaccines, microbicides, and PrEP [7,30,31]. Additionally, MSM expressed concerns about stigma if it were disclosed that they were accessing these HIV prevention products, such as being judged as sexually “promiscuous” or being “outed” to their families as MSM. This invoked the potential importance of the venue for accessing HIV prevention products as a determinant of product acceptability and use.

Second, we conducted a pilot DCE study with MSM (n = 16) recruited from CBO partners in India; this provided a priori guidance on the model coefficients and guided the selection of attributes (e.g., efficacy) and levels (e.g., 50% vs 99%) for each product in the DCE [32]. It also supported the feasibility of implementing DCEs with this population.

Third, we incorporated methodological recommendations for choice elicitation tasks [32]: alternative attribute levels (e.g., 50% vs 99% efficacy; none vs. minor side effects) need to be sufficiently distinct to be comprehensible to participants, most of whom are unlikely to be able to discern the difference between 50% and 65% efficacy, for example, or a 15% vs. 25% chance of fever or headaches. Our recruitment aimed to reach vulnerable populations of largely low socioeconomic status MSM, many without college education and with limited economic opportunities, a substantial proportion of whom rely on sex work for income. This underscored the importance of selecting attribute levels to facilitate comprehension among participants with low numeracy.

Finally, the very high efficacy of oral PrEP in clinical trials informed our use of 99% as a level. As a result, 99% efficacy was used as the upper value for all 3 products, in contrast with 50% to clearly signify partial efficacy.”

Limitations section (page 2, paragraph 3):

“It is also an inherent limitation of DCEs that the preferences revealed are contingent on the product attributes and values modeled. The present study’s use of categorical variables with two levels of each attribute was based on formative qualitative research and pilot DCEs with the study population, following best practices in the development of the DCEs. Although relevant attributes and levels of products in the development pipeline may change as they near approval, this is commensurate with our aim to prepare for future implementation of HIV prevention products in the development pipeline, a benefit of DCE methods.”
